# Effect of Recombinant Insulin-like Growth Factor-2 Injected into the Hippocampus on Memory Impairment Following Hippocampal Intracerebral Hemorrhage in Rats

**DOI:** 10.22086/gmj.v0i0.1353

**Published:** 2018-11-19

**Authors:** Farzaneh Vafaee, Asadollah Zarifkar, Masoumeh Emamghoreishi, Mohammad Reza Namavar, Marzieh Shahpari, Amir Hossein Zarifkar

**Affiliations:** ^1^Department of Neuroscience, School of Advanced Medical Sciences and Technologies, Shiraz University of Medical Sciences, Shiraz, Iran; ^2^Department of Physiology, School of Medicine, Shiraz University of Medical Sciences, Shiraz, Iran; ^3^Shiraz Neuroscience Research Center, Shiraz University of Medical Sciences, Shiraz, Iran; ^4^Department of Pharmacology, School of Medicine, Shiraz University of Medical Sciences, Shiraz, Iran; ^5^Clinical Neurology Research Center, Shiraz University of Medical Sciences, Shiraz, Iran; ^6^Histomorphometry and Stereology Research center, Shiraz University of Medical Sciences, Shiraz, Iran; ^7^Department of Anatomical Sciences, School of Medicine, Shiraz University of Medical Sciences, Shiraz, Iran

**Keywords:** Intracerebral Hemorrhage, IGF-2, Learning, Memory, Hippocampus

## Abstract

**Background:**

Insulin-like growth factor 2 (IGF-2) is a growth factor and an anti-inflammatory cytokine that plays a pivotal role in memory. In this study, we examined the effect of recombinant IGF-2 on memory impairment due to intracerebral hemorrhage (ICH). Avoidance and recognition memory, locomotor activity, neurological deficit score (NDS), and the level of the IGF-2 gene expression were evaluated.

**Materials and Methods:**

To induce ICH, 100 μL of autologous blood was injected into the left hippocampus of male Sprague Dawley rats. Recombinant IGF-2 was injected into the damaged hippocampus 30 minutes after the induction of ICH. Then, over two weeks, NDS, locomotor activity, passive avoidance, and novel object recognition (NOR) test were evaluated. Finally, the level of IGF-2 gene expression was evaluated by using the real-time polymerase chain reaction technique.

**Result:**

Our results indicated that recombinant IGF-2 injection significantly increased step-through latency (P<0.001) and total time spent in the dark box (P<0.01). However, no significant difference was seen in recognition memory and NDS. Locomotor activity did not significantly change in any group. A significantly reduced level of IGF-2 was observed after two weeks (P<0.05).

**Conclusion:**

The results of this study show that a single dose of recombinant IGF-2 injection can influence hippocampus-dependent memories. Importantly, IGF-2 did not change locomotor activity and NDS after two weeks, which probably represents its specific function in memory.

## Introduction

Memory impairment is a debilitating factor in many diseases such as stroke, trauma, neuroinflammatory, and neurodegeneration diseases; it is also time-consuming and costly to treat in today’s society [1]. In hemorrhagic stroke, blood pressure or trauma causes blood vessel rupture, intracerebral hemorrhage (ICH), and brain damage. When this damage is in the hippocampus, it causes disabling memory disorder in patients [2,3]. ICH can damage the brain through driven complications of inflammation, upregulated neutrophil infiltration, glial activities, local cytokine imbalance, and oxidative stress [3,4]. Among these, inflammation plays a key role in developing secondary brain damage. Many studies have focused on the anti-inflammatory factors involved in the treatment of stroke [5]. In the line of finding potent approaches to improve memory impairment, strategies affecting the transcription factor cAMP-responsive element-binding protein (CREB) through medications are of great importance [6]. CREB is activated by various signaling pathways such as growth factors, activation of N-methyl-D-aspartate receptors, and other neurotransmitters. CREB also activates transcription-targeted genes that play a role in memory enhancement [7-9]. The activation of these genes leads to increased learning and expression of growth factors and anti-inflammatory factors, such as insulin-like growth factors, in the hippocampus [10]. Therefore, promoting an effective drug that can specifically affect the activity of genes involved in memory, reduce inflammation, act as a growth factor, and speed up the repair with minimal side effects is very important.

Insulin-like growth factor 2 (IGF-2) is a polypeptide that belongs to the insulin family [11]. Studies have shown that IGF-2 plays a key role in enhancing cognitive and memory activities [10]. The activation of IGF-2 receptor causes responses such as regulating calcium homeostasis, increasing acetylcholine secretion, and ultimately proliferating cells and helping them survive [12]. IGF-2 can act as an effective medication for regulating calcium homeostasis and preventing neurotoxicity and can play a role in increasing memory [13-15]. Schmeisser showed that IGF-2 could increase the density of the dendritic spine, and it also plays a role in the formation and regulation of synapses [16]. Lipopolysaccharides (LPS), a type of proinflammatory factor, can increase IGF-2 in microglial cell cultures; in addition, the results of real-time polymerase chain reaction (PCR) analysis and western blot indicate that IGF-2 significantly increases after inflammation, illustrating that IGF-2 plays a key role as an anti-inflammatory factor [17,18]. In studies conducted by Alberini et al. on healthy mice showed that injecting IGF-2 could activate the CREB pathway and improve memory. Prior to these studies, it was thought that IGF-2 was most important as a growth factor in evolution. However, Alberini et al. showed that IGF-2 is not only important in the adult brain but also plays a key role in memory consolidation [1,7,9,10].

Although studies show that IGF-2 can increase memory in healthy mice and improve memory in neurodegenerative diseases such as Alzheimer disease, we could not find any study about the effect of IGF-2 on neuroinflammation diseases and acute brain injuries such as stroke or trauma. Moreover, the effect of IGF-2 on memory impairment in pathologic conditions and brain damage is still unknown. Our hypothesis in this study is that the injection of exogenous IGF-2 into the hippocampus after brain injury following ICH is effective in memory consolidation and retention. In this study, we injected IGF-2 into the injured hippocampus 30 minutes after induced ICH and performed behavioral tests after two weeks. Next, using molecular studies, we examined the level of the IGF-2 gene expression.

## Methods and Materials

### 1. Animals

This study included 24 adult male Sprague Dawley rats weighing 220 to 250 g that were acquired from the Center of Comparative and Experimental Medicine at Shiraz University of Medical Sciences, Iran. The rats were housed in accordance with the standard conditions of ambient temperature (22 ± 2°C), 12-12–hour light-dark cycle, and free access to water and food. The guidelines proposed by National Institutes of Health Guide for the Institutional Animal Care [19] and Institutional Ethics Committee at Shiraz University of Medical Sciences (ethical code: IR.SUMS.REC.1395.S767) were applied to perform experiments. Animals were handled five days before the behavioral experiments. On the day of the test, they were placed in the laboratory half an hour before starting the experiment to help them adapt to the environment.

### 2. Blood Samples Collection

Following general anesthesia, blood samples were obtained from the rats using a capillary tube inserted into the medial canthus of the eye, with 30° toward the nose. The tissue was punctured with thumb pressure to insert the plexus/sinus for holing blood flow into the capillary tube and taking samples. After removing the capillary tube, sterilized cotton was used to clean, and finger pressure was applied to prevent bleeding. After incubating the collected blood sample at room temperature (15 minutes), it was injected into the hippocampus [20].

### 3. Surgical Procedure

The animals were anesthetized with ketamine (100 mg/kg, i.p.) and xylazine (10 mg/kg, i.p.), and then, they were located in a stereotaxic frame for ICH surgery. A 2-cm incision was made in the shaven head skin to remove all soft tissue on the skull before drilling. According to Paxinos and Watson [21], a 26-G Hamilton needle (100 µl, 700 series, Hamilton Company, Switzerland) was placed in the left hippocampus in the coordinates of 3.4-mm anterior (AP), 2.4-mm lateral (ML), and 3.6-mm ventral (DV) in relation to Bregma [22]. Autologous blood (100 µL) the orbital sinus was injected unilaterally into the left hippocampus gently within 5 minutes. The blood starts to clot after 10 minutes, and the clot should be removed to impede the backﬂow.

Acrylic dental cement was used to seal the drilled hole, and the skin was sutured. Meloxicam (0.1 mg/kg) was injected to maintain the postoperative analgesia. The sterilized saline (vehicle) was replaced with blood in the sham group, and 250 ng/µL IGF-2 (R&D, USA), according to our pilot study, was injected into the hematoma, 30 minutes after injecting the blood in the ICH-IGF-2 group. After the end of the surgical procedure, the operated rats were housed aseptically again in the same cage and controlled until they recovered from anesthesia [23-25].

### 4. Behavioral Test

#### 4.1. Neurological Deficit Score (NDS)

All six parameters of the NDS, including body symmetry, climbing, gait, circling behavior, compulsory circling, and front limb symmetry, were measured after 1, 3, 7, and 14 days of post-ICH for each rat. Each item was scored from 3 to 18, with three referring to the maximum deficit and 18 to normal [26].

#### 4.2. Assessment of the Wire-Hanging Test

A wire (2 × 60 mm2) was installed between 2 platforms with a height of 50 cm, and then the animals were placed midway for a maximum of 5 minutes. In this way, the grip strength and balance were observed on days 1, 3, 7, and 14 post-ICH. To control any injury caused by possible falls, a pillow was placed under the animal. The suspension time of the rat on the wire was recorded [27,28].

#### 4.3. Novel Object Recognition (NOR) Task

An open ﬁeld apparatus (72 × 72 × 35 cm3) with a white wall was used to test the behavioral function. Each animal was left in the box for 5 minutes for the first time to familiarize with the environment without any stimuli after 14 days of induced ICH. The following day, the rats were trained for 10 minutes through 2 identical objects (A1 and A2). After an hour of rest, they were allowed to be familiar with 1 of either object A1 or A2 in the box, or 1 novel object B with different shape and color for 5 minutes. The total time of exploring the novel and old objects was recorded for each animal, including time in direct contact and time within the object area. If the nose of the animal was directed at the object at a distance of less than 2 cm, the rat was regarded as in the object area. The recognition memory sensitivity is evaluated by using the discrimination ratio (DR). DR indicates the time spent next to the new object compared with the time spent next to the old object, which is reported as a percentage of the total time [29,30]. DR is calculated as

DR=(T(new)-T(old))/(T(total))

#### 4.4. Passive Avoidance Test

The rats underwent passive avoidance training on day 14 after surgery, using a shuttle box with two connected chambers of equal size separated by a guillotine door. All animals were initially left in the apparatus to be familiar with the environment without any stimuli. The control and experimental groups were guided individually into the lighted chamber for 10 seconds. The installed door was opened to note the latency to enter the dark chamber. If any animal did not enter the darkness within 60 seconds, it was replaced with a new rat. After 5 minutes, the habituation step was repeated for the same interval. The third adaption trial was conducted after 2 hours where the animals’ feet were exposed to an electrical stimulation (0.5 mA, 50 Hz, 2 seconds once) delivered through the stainless steel floor in the dark chamber for 20 seconds. Next, the animals were returned to their own cage. This test was repeated after 5 minutes. In this way, the successful acquisition of passive avoidance response was considered in case the animals avoided entering the darkness up to 300 seconds.

After a day, the rats were re-entered into the illuminated chamber for the retention trial, without any foot shock. After entering into the darkness, the latency to re-enter the dark chamber and total time spent in the dark box were recorded. The time of going into the dark compartment was regarded as the step-through latency (STL) in the two learning and retention trials. As mentioned, the maximum cut-off time for the STL was 300 seconds in the retention test [31,32].

### 5. IGF-2 Gene Expression

#### 5.1. Collection of Tissue to Perform Real-Time PCR

Once the animals were decapitated, the brains were separated immediately. Their hippocampi were isolated from the left hemisphere immediately and were frozen using liquid nitrogen inside microtubes and kept at a temperature of –80°C.

#### 5.2. Extraction of RNA

A homogenizer handle was used to homogenize the samples that were then incubated in ice for 5 min. Afterward, the homogenates were centrifuged for 15 minutes at 12,000 × g at 4°C. To isolate RNA, 1 mL of TRIzolTM reagent (Invitrogen^TM^, São Paulo, Brazil) in 5 parts of 200 µL was appended for five times for a total of 50 to 100 mg of tissue. The mixture was shaken for 15 minutes each time. To completely dissociate the nucleoprotein complex, the homogenized samples were incubated for 5 minutes at the RT. Then, 250 µL of chloroform was added for 1 mL of TRIzol reagent.

The tubes were completely shaken for 15 seconds and then were incubated at the RT for 2 to 3 minutes. The samples were centrifuged for 15 minutes at 12,000 × g at 4°C, and 0.4 mL of the obtained aqueous phase was poured into another tube. Precipitation was achieved by 0.4 mL of cold isopropanol per 1 mL of the TRIzol reagent. The samples were incubated over 10 minutes at RT and then centrifuged for 10 minutes at 12,000 × g at 4°C. Afterward, the samples were incubated overnight at –20°C.

The supernatant was discarded, and the pellet was rinsed once with 1 mL of 75% diethylpyrocarbonate (DEPC)–treated ethanol in the presence of 1 mL of the TRIzol reagent. The pellet-containing RNA was air-dried for 5 to 10 minutes and subsequently with a hot block for 1 to 2 minutes.

Next, the pellet was resuspended in the DEPC-treated water. A spectrophotometer (λ260, ultraviolet light) was used to measure the quantity of RNA, and 1 μL of RNA was diluted in 99 μL of DEPC-treated water. The stock concentration was obtained on the basis of 1 OD = 40 μg/mL of RNA and the dilution of 1/100. RNA was kept at –80°C.

#### 5.3. Synthesis of cDNA

All-in-One^TM^ First-Strand cDNA Synthesis Kit (GeneCopoeia, Inc., USA, Cat. No. AORT-0050) was applied to synthesize the cDNA; thus, total RNA (1 μg) was considered as template. The incubation of the final volume of 13-μL RNA was performed in the presence of 1 μL of oligo (dT), 1 μL of random primer, and double-distilled water (ddH2O; RNase/DNase-free) at 65°C for 10 minutes, followed by an instant temperature drop on ice. Next, the final volume of 25 μL was set using RNase/DNase-free ddH2O, 13 µL of RNA-Primer Mix, 5 µL of RT Reaction Buffer, 1 µL of dNTP, 1 µL of RNase inhibitor, and 1 µL of M-MLV RTase. The resulting reaction mix was incubated at 37°C for 60 minutes. Finally, the reaction process was ended by exposing it to 85°C for 5 minutes and kept at –20°C until testing.

#### 5.4. Real-Time PCR

The StepOnePlus^TM^ thermocycler (Applied Biosystems^TM^, Foster City, CA, USA) was used to process the reactions in a 96-well plate containing PCR master mix (Applied Biosystems^TM^, São Paulo, Brazil) of SYBR^TM^ green (12.5 μL), forward and reverse primers (0.5 μL each), cDNA (100 ng), and the rest nuclease-free water in a total volume of 25 μL.

PCR primers were designed with Gene Runner (Version 6.5.51, free online Software, Inc., Hudson, USA) online software and controlled by the Basic Local Alignment Search Tool to ensure no cross-reactivity.

In all reactions, the housekeeping β-actin gene was run separately with the same experimental conditions to obtain the RNA integrity and quantity in the onset of real-time reaction. The rat IGF-2 (forward sequence of 5′-TGTCATTGCTTCAGTGCTCTCT-3′ and reverse sequence of 5′-TTCTGTTCCTCTCCTTGGGTTC-3′ with the product size of 163 bp) and rat GAPDH (forward sequence of 5′-AAGTTCAACGGCACAGTCAAGG-3′ and reverse sequence of 5′- CATACTCAGCACCAGCATCACC-3′ with the product size of 121 bp) were the primers used in the PCR process.

The protocol of the PCR procedure was denaturation at 95°C for 15 minutes, 50-cycle amplification with denaturation (95°C, 15 s), annealing (60°C, 60 s), and extension (72°C, 60 s). The specificity of PCR products was determined by a melting curve analysis. The algorithm enhancements provided by the equipment estimated the experimental threshold cycle (Ct). All samples were tested twice, and the calculated mean values were analyzed. The Ct values were achieved with the aid of instruments in each reaction using default parameters. The reactions were separately continued in the tubes for the same samples to test the levels of IGF-2 and β-actin. The equation of 2–ΔΔCt was used to calculate the relative quantiﬁcation of the IGF-2 mRNA expression levels.

### 6. Statistical Analysis

Data for each group were analyzed using the SPSS 16.0 software (SPSS Inc., IBM) and described using graphs, means, and standard error of the mean. The Kolmogorov-Smirnov test was used to determine the normal distribution of data, and the one-way ANOVA with the Tukey-Kramer post hoc test was used to fulfill the intergroup comparison. To analyze NDS statistically, the nonparametric Mann-Whitney test was applied. In all tests, the statistical significance level was P < 0.05.

## Results

### 1. Behavioral Assessment Test

#### 1.1. Effects of IGF-2 on Neurological Analysis and Locomotor Function

**Figure 1:**
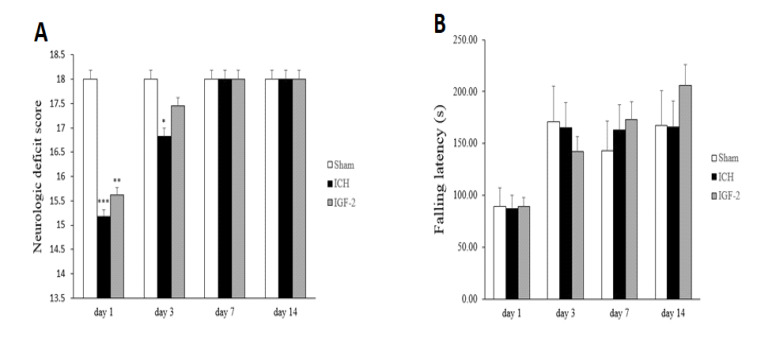
**(A)** IGF-2 effect on NDS. Data are shown as mean ± SEM (n = 8). * P <0.05, **P < 0.01, and ***P <0.001 vs. sham group; **(B)**GF-2 effect on locomotor deficits evaluated by the wire-hanging test. There was no significant difference between groups.

We measured the NDS using an 18-point scoring system in which lower scores mean greater deficit. The motor deficit was significantly higher in the sham rats than in the ICH group (15.17±0.26, 16.83 ± 0.40, respectively) in days 1 and 3, and significantly lower in the IGF-2 treatment group (15.64 ± 0.31) than in the sham group just in day 1 (Figure 1A). The wire-hanging test indicating the grasping for forelimb strength and motor coordination. In this test, rats hold on to the wire with their forelimbs. Based on the findings, no significant differences in time spent on the wire were found among all groups during 1, 3, 7, and 14 days after surgery (Figure 1B).

#### 1.2. IGF-2 Effects on Passive Avoidance Learning

**Figure 2:**
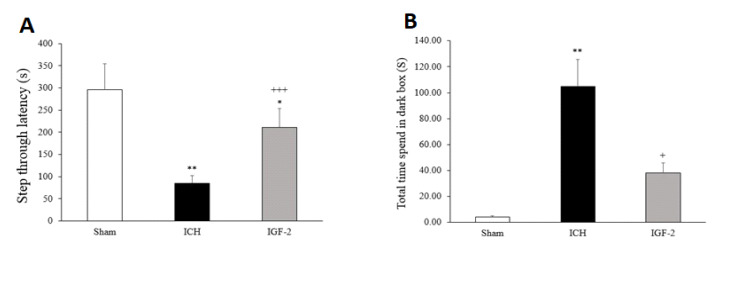
**(A)** Effect of IGF-2 on STL in shuttle box test. Data are shown as mean ± SEM (n = 8). *P < 0.05 and ***P < 0.001 vs. sham group; +++ P < 0.001 vs. ICH group; **(B)** Effect of IGF-2 on TDC in the shuttle box test. **P < 0.01 vs. sham group; +P < 0.05 vs. ICH group

Based on the passive avoidance test results, 1 or 2 ft shocks made the rats in all groups to learn the avoidance task. No statistical difference was seen in the training trials (data not shown). IGF-2 treatment significantly prolonged the STL time and improved memory retention (211.43 ± 29.31) compared with the control (85.75±14.01). Further, time in dark compartment (TDC) was significantly higher in ICH group (104.63 ± 24.47) than in the sham group (4.29 ± 4.29). Also, TDC was significantly less in the treatment group (38.14 ± 13.07) than the control group (Figure 2).

#### 1.3. IGF-2 Effects On NOR Test in Assessing Learning and Memory

The NOR test was used to evaluate recognition memory. According to the data obtained, the discrimination ratio of the test, the duration of the object investigation by the rats in the training session was not found to be significant (data not shown).

**Figure 3:**
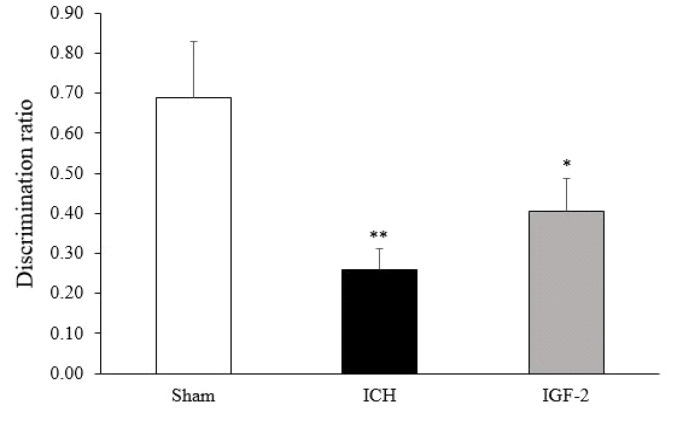
Effect of IGF-2 in NOR test. Data are shown as mean ± SEM (n = 8). In contrast to the sham group, rats in ICH and IGF-2 groups spent less time exploring novel object than old ones. No significant difference was observed between the IGF-2 and ICH groups. *P<0.05 and **P<0.01 vs. sham group.

The ICH group (0.26 ± 0.062) had less exploration time in the novel object than the sham group (0.69 ± 0.078). Further, time was spent by the IGF-2 group to seek the novel objects (0.41 ± 0.089). However, this did not show any significant difference compared with ICH groups (Figure 3).

### 2. Decreased Expression of IGF-2 Gene Due to Recombinant IGF-2 Injection

**Figure 4:**
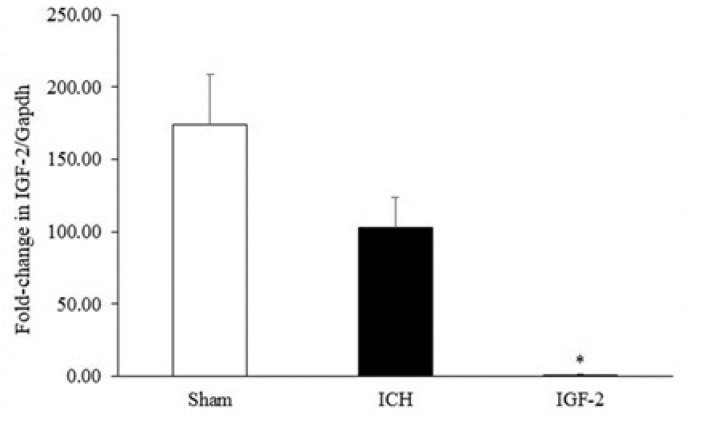
Effect of recombinant IGF-2 on the level of the IGF-2 gene expression in a damaged hippocampus following ICH. The mRNA level of IGF-2 measured using the real-time PCR technique was normalized by GAPDH. Data are shown as mean ± SEM (n = 4). *P<0.05 vs. sham group.

The level of IGF-2 mRNA in the hippocampus was measured using the real-time PCR technique (Figure 4). All study groups exhibited the expression of IGF-2, but the IGF-2 mRNA level was significantly reduced (1.6 ± 0.91) after injecting the 20 ng of IGF-2 into the left hippocampus. IGF-2 mRNA was obvious in the ICH group (103.22 ± 30.25), but there was no significant difference with the sham group (174.12 ± 44.48), suggesting the reduced expression of IGF-2 due to ICH-induced brain damage and suppressed expression of IGF-2 in the presence of higher exogenous IGF-2.

## Discussion

In this study, we observed that recombinant IGF-2 could significantly increase avoidance memory, but did not have a significant effect on recognition memory impairment caused by ICH. However, our data are in line with studies by Alberini et al. that suggest IGF-II administration—both intrahippocampal and systemically—significantly improves memory types [1,9,10]. In a study conducted by Pascual-Lucas et al. on a transgenic mouse model showed that IGF-2 could improve memory and reduce synaptic deficiency [33]. To our knowledge, there is no any other study on the effect of IGF-2 on memory impairment due to acute cerebral injury, such as stroke and trauma. Considering that IGF-2 is a growth factor and a potent anti-inflammatory factor without any reported adverse effect, it can be considered an effective treatment.

The IGF-2 connection to its receptor activates the downstream PI3K/Akt pathway. This pathway affects the growth and survival of neurons. Also, IGF-2 induces an upregulated C/EBPβ target gene, which plays a key role in memory stability. Studies have shown that training increases the release of IGF-2, and this increase persists for up to 4 days after training [1,10,34]. IGF-2 plays a key role in hippocampus-dependent memories. Another reason for the effectiveness of IGF-2 in memory enhancement is the expression of the activity-regulated cytoskeletal protein (Arc) following the activity of the IGF-2 receptor. Arc—a protein with an important role in learning and memory-related molecular process—was induced by synaptic activity [10,35].

In this study, we observed that a single-dose administration of IGF-2 significantly improved avoidance memory but not recognition memory, although the trend was increasing. Our results are contradictory to the study by Steinmetz et al., which showed that using IGF-2 caused increase memory in both averse and nonaversive memories in an aged rat [36]. Stern et al. showed systemic IGF-2 change recognition memory in healthy mice [1], and Lee et al. reported hippocampal IGF-2 injection after training-enhanced recognition memory [37]. Both passive avoidance and NOR tests evaluate hippocampus-dependent memories. However, there are differences in the retention and consolidation pathways between these tests, which can be suggested as a possible reason for our results. It is worth mentioning that previous studies did not investigate the effect of IGF-2 in acute brain injury; thus, a single dose of IGF-2 injection is probably not enough to improve recognition memory.

IGFs are secreted as endogenous anti-inflammatory factors of M2 microglia after injury [17,38]. Human culture studies have shown that the main source of IGF-2 is microglia. Also, IGF-2 production has been shown to increase following the upregulation of inflammatory factors such as IL-4 and IL-13. Increasing LPS in the cell culture also increases the production of IGF-2 [17,39,40]. This study shows that IGF-2 is elevated in response to inflammation and works as a potent anti-inflammatory factor. In this study, IGF-2 has not caused significant change in NDS. It is likely that the inflammatory factors have changed, but this difference has not been sufficient to affect the NDS. Also, in the NDS, motor and sensory systems are evaluated, where the most impact of IGF-2 is on memory. The probable part of the mechanism for improving memory after ICH is related to its function as an anti-inflammatory factor.

The IGF-2 receptor activates the CREB pathway, which has been shown to be involved in synaptic plasticity and memory formation. The CREB-C/EBP cascade enhances the expression of the IGF-2 gene and subsequently increases IGF-2 levels in the environment [9,41]. Our results showed that the expression of the IGF-2 gene in the IGF-2 group was significantly decreased in comparison with the sham group. IGF-2 works as a growth factor; thus, in addition to cell survival, overexpression of IGF-2 can cause an increase in cancer cells [41,42]. The brain has defensive mechanisms that prevent the excessive function of this factor and its receptor. One of these mechanisms is increased expression of miRNAs that are responsible for regulating IGF-2 gene expression. By increasing the activity of this miRNAs, IGF-2 is downregulated, and IGF-2 gene expression is inhibited [43]. The reduction of IGF-2 gene expression in the treatment group can be due to the possible increase of IGF-2 levels in the tissue and the probable subsequent suppression in the expression of the IGF-2 gene.

Stern et al. investigated the effect IGF-2 in various parameters and reported IGF-2 did not have any side effect in her study [1]. We observed that locomotor activity did not change in any group, which represents that IGF-2 did not affect motor activity and coordination. Hence, our results somewhat confirm the results of previous studies.

## Conclusion

In summary, the results of this study indicate that the injection of recombinant IGF-2 into the hippocampus 30 minutes after the ICH can improve avoidance memory. However, it does not have a significant effect on recognition memory. Also, IGF-2 was not effective on motor activity assessed by the wire-hanging test, which indicates that IGF-2 worked specifically on memory. Even though IGF-2 injection led to the improvement of memory, it reduced the IGF-2 gene expression after two weeks, which requires further studies.

## Conflict of Interest

Authors have no conflict of interests.
